# Acknowledgments

**DOI:** 10.4269/ajtmh.1103siii

**Published:** 2024-03-05

The authors would like to express their deepest appreciation to: National Malaria Program staff in all PMI Impact Malaria-supported countries for their leadership and sharing the results and lessons learned from their successful programs; All health workers, supervisors, and district and regional staff who contributed to improvement in health services documented in this supplement; Ricki Orford, Mary Warsh, and Natalie Hendler for their leadership of PMI Impact Malaria; Lawrence Barat for his technical leadership of PMI Impact Malaria and leadership and coordination in the development of this supplement; All PMI Impact Malaria headquarters and country staff for their support of the country activities that are reported in this supplement; Gretchen Newby for her pivotal editorial and capacity building support to the authors; Taylor Prochnow and Timour Razek for their support of these publications; S. Patrick Kachur for his editorial oversight; Kim Connolly, Meera Venkatesan, Kevin Griffith, Jordan Burns, Delissa Jackson, Shawn Kerry, Annē Linn, Lia Florey, Rose Zullinger, and Ashley Malpass for their oversight of PMI Impact Malaria and for critical review of the manuscripts.

Financial support: Funding for this supplement was provided by the U.S. President’s Malaria Initiative.

Disclosure: The contents of this supplement are the responsibility of the authors and do not necessarily reflect the views of the United States Agency for International Development or the U.S. government.

